# Social Determinants and Differences in Mortality by Race/Ethnicity

**DOI:** 10.1001/jamanetworkopen.2019.21392

**Published:** 2020-02-05

**Authors:** Eliseo J. Pérez-Stable, Erik J. Rodriquez

**Affiliations:** National Institute on Minority Health and Health Disparities, National Institutes of Health, Bethesda, Maryland; Division of Intramural Research, National Heart, Lung, and Blood Institute, National Institutes of Health, Bethesda, Maryland

Race, ethnicity, and socioeconomic status are strongly associated with many health and health care outcomes; thus, these factors should always be considered as demographic variables in evaluating population health. The National Institute on Minority Health and Health Disparities has created a framework to guide scientists in defining multilevel pathways and interventions to address these questions.^1^ The findings reported in the study by Spillane and colleagues^2^ generate questions to be addressed in population health.

The observation that overall mortality for African American individuals has been decreasing over the past 2 decades^3^ has not received nearly the amount of attention that has been received by the increase in overall mortality over the past several years in the United States. In fact, the mortality gap between African American individuals and white individuals has been eliminated for individuals 65 years and older since 2010.^3^ Understanding the factors associated with this remarkable accomplishment in population health is an important component of minority health science.

At-risk alcohol use, defined as excessive daily drinking or periodic episodes of binge drinking, has generally been more prevalent among racial and ethnic minority groups and individuals who are economically disadvantaged. Mortality from alcohol use, as captured by death certificates, is driven by chronic liver disease, accidental poisoning, and mental or behavioral disorders. African American individuals have a higher risk of death from chronic liver disease and liver cancer compared with white individuals. The findings of Spillane et al^2^ suggest that other causes, such as chronic hepatitis, may be underlying factors. On the other hand, African American individuals also have an unexplained lower risk of completed suicide, which has remained stable over the past 2 decades despite suicide being one of the causes of death associated with the overall increase in deaths of despair in the United States.^4^

Understanding the factors associated with why a disadvantaged population has a lower risk of mortality from specific causes is an important research question. Leveraging available national data on known risk factors, differential coping with stress among African American individuals vs white individuals has been proposed by the environmental affordances model.^5^ In that study, Jackson et al^5^ argue that the higher rates of suicide among white individuals were associated with a higher rate of depression given this group’s response to stress. By comparison, African American individuals had less depression but higher rates of substance use and unhealthy eating. Other analyses examining Latino individuals have not supported this association using either self-reported measures of chronic stress or allostatic load score.^6,7^ Research is needed that stratifies populations by race and ethnicity to test whether hypothesized mechanisms function the same ways in different population groups.

Evaluation of alcohol-induced deaths in this report should not diminish the importance of other alcohol-related morbidity. A substantial proportion of unintentional injuries, including motor vehicle crashes, firearm violence, and social disruption within families and communities, are associated with at-risk alcohol use. These events are associated with long-term outcomes that can contribute to chronic stress and poor health outcomes, and they occur more frequently among African American individuals.^8,9^ Despite the elimination of the mortality disparity for African American individuals at age 65 years, significant disparities in quality of life and chronic disease persist.

Finally, on a cautionary note, although alcohol-induced deaths decreased overall for African American individuals, recent trends from 2012 to 2016 in men and from 2007 to 2016 in women showed concerning increases. It is possible that these data reflect a greater effect of external factors, such as the Great Recession and higher rates of alcohol use, in younger cohorts of African American individuals compared with older African American individuals. A 2020 study analyzing mortality data^10^ showed this same increase in recent years. Additional granular data on geographic variation among African American individuals would further inform these analyses. Continuing this surveillance and analysis to evaluate societal trends is critical as clinicians and scientists attempt to modify the underlying factors associated with alcohol-induced morbidity and mortality. Addressing these questions using race, ethnicity, and socioeconomic status as essential categories to evaluate outcomes would enrich our understanding and inform the development of effective interventions.
